# Design of highly efficient phosphor-converted white light-emitting diodes with color rendering indices (*R*_1_ − *R*_15_) ≥ 95 for artificial lighting

**DOI:** 10.1038/s41598-019-53269-0

**Published:** 2019-11-14

**Authors:** Yong Nam Ahn, Kyu Do Kim, Gopinathan Anoop, Gab Soo Kim, Jae Soo Yoo

**Affiliations:** 1 School of Chemical Engineering and Materials Science, Chung-Ang University, Seoul, 06974 South Korea; 2 LED division, R&D Team, Daejin DMP Co., Ltd, Chungchengnam-do, Cheonan, 31044 South Korea; 3 School of Materials Science and Engineering, Gwangju Institute of Science and Technology, Gwangju, 61005 South Korea; 4 Kimin Electronics Co. Ltd, Gumi-Si, Gyeongsangbuk-do 39378 South Korea

**Keywords:** Inorganic LEDs, Inorganic LEDs, Chemical engineering

## Abstract

Phosphor-converted white light-emitting diodes (pc-WLEDs) are excellent energy-efficient light sources for artificial lighting applications. One goal of artificial lighting is to make objects/images look natural – as they look under the sunlight. The ability of a light source to accurately render the natural color of an object is gauged by the parameter – color rendering index (CRI). A conventional pc-WLED has an average CRI ~ 80, which is very low for accurate color reproduction. To utilize the pc-WLEDs for artificial lighting applications, all the CRI points (*R*_1_ – *R*_15_) should be above 95. However, there is a trade-off between CRI and luminous efficacy (LER), and it is challenging to increase both CRI and LER. Herein we propose a novel LED package (PKG) design to achieve CRI points ≥95 and efficiency ~100 lm/W by introducing two blue LEDs and a UV LED in combination with green and red phosphors. The silicone encapsulant, the current through the LEDs, and the green/red phosphor ratio were optimized for achieving high CRI and LER. Our re-designed LED PKG will find applications in stadium lighting as well as for ultra-high-definition television production where high CRI points are required for the artificial light source.

## Introduction

Phosphor-converted white light-emitting diodes (pc-WLEDs) have emerged as next-generation solid-state lighting technology and is a potential substitute for traditional lighting sources such as incandescent lamps, fluorescent lamps, halogen lamps, and backlights for liquid crystal displays^1–3^. The low power consumption, high luminous efficacy, and environment-friendly characteristics make the pc-WLEDs attractive. The most commonly accepted and commercially available pc-WLEDs are based on the combination of a blue-chip and yellow phosphor or blue-chip, green, and red phosphors. Our group and others have studied various phosphors and utilized the phosphors for fabricating highly efficient and reliable WLEDs^4–13^. Several strategies have been proposed to enhance the luminous efficacy and stability of the pc-WLEDs^14–17^. However, apart from the high luminous efficacy, accurate color reproduction is also a major requirement when the WLEDs are used as an artificial light source, especially when WLEDs are used for photography and videography. The color rendering index (CRI)– defined by Commission Internationale de l'éclairage (CIE, International Commission on Illumination) is a quantitative measure that provides the ability of a light source to reproduce the color of the object it illuminates accurately^18,19^. The standard CRI points, namely *R*_1_ − *R*_8,_ are derived from CIE 1974 test color samples and usually arithmetical mean of these values – *R*_*a*_ is used to report the ability to reproduce the color accurately. The special CRI points (*R*_9_*- R*_15_) that were derived from realistic colors are also used as a color rendering marker. The recommended *R*a values depend on the application environment; nevertheless, *R*_*a*_ of > 80 is recommended for most applications. For lighting in stadiums, most of the athletic committees recommend *R*_a_ > 90. Even though *R*_*a*_ is used to evaluate the light sources, saturated colors cannot be evaluated accurately in terms of *R*_*a*,_ and numerous new methods for color rendition evaluation have been proposed. Among the new methods, technical memorandum (TM-30) developed by Illuminating Engineering Society (IES) uses 99 color samples, including saturated colors^20,21^. In the TM-30 standard, fidelity index (*R*_f_), gamut index (*R*_g_), color vector/saturation graphics, hue fidelity indices (*R*_f,h*j*_, j = 1 to 16), chroma change by hue indices (*R*_cs,h*j*_, j = 1 to 16), skin fidelity index (*R*_f,skin_) and sample fidelity index (*R*_f,CES*i*_, i = 1–99) are used to evaluate a light source. Depending on the application environment, these values vary and are evaluated as per the specific requirements. It is crucial to have a decent color quality with reasonable efficacy suitable for intended applications. Unlike incandescent and fluorescent lamps, the light-emitting diodes (LEDs) emit light in a narrow range of wavelengths in the visible spectrum. This unique optical characteristic has offered big potential for LEDs to produce high-quality white light with reasonable luminous efficacy by combining proper inorganic phosphors. The LEDs as a light source are now far beyond from being a promising technology, and the market share of the LEDs in the field of general illumination is dramatically increasing due to the low manufacturing cost. Also, applications of LEDs are extended to new fields such as horticulture, biologically-driven lighting, and lighting for the art exhibition^22–24^. For each application, the requirement varies, and the LEDs must be properly evaluated to find the suitability as per the requirement. Concerning the application environment, rather than fabricating LEDs with CRI *R*_a_ >80 or 90, individual CRI points (*R*_1_- *R*_15_) should be evaluated in detail. In this aspect, recently, a combination of two CRI points such as *R*_a_ and *R*_9_ of light sources are evaluated, and most of the LED manufacturers label *R*_a_ and *R*_9_ on the product specifications. Even if *R*_a_ >80, the *R*_9_ values will be low (<10) for conventional WLEDs consisting of a blue-chip and yellow phosphor. Because *R*_9_ corresponds to the red component and the blue LED -yellow phosphor combination in WLEDs lack red component. Moreover, for ultra-high-definition television (UHDTV) production, high CRI points (>95) of the light source is required. Therefore, conventional WLEDs consisting of a blue LED and yellow phosphor should be replaced with a high CRI LED for such specific applications. However, it is challenging to enhance CRI points and luminous efficacy simultaneously as there is a trade-off between them^25^. In this work, we have re-designed and optimized the LED package design parameters with high luminous efficacy and all the CRI points above 95 by introducing a combination of two blue LEDs and a UV LED along with green and red phosphors. Through properly optimizing the current through each LED and the phosphor ratio, we achieved all the CRI *R*_1_ – *R*_15_ values above 95 and luminous efficacy around 100 lm/W. The LEDs were compared with the conventional blue LED - yellow phosphor, blue LED - green & red phosphors, and blue LED - blue, yellow, and red phosphors based LEDs. All the LEDs were evaluated using the TM-30-18 standard and compared.

## Results and Discussions

**Figure 1:**
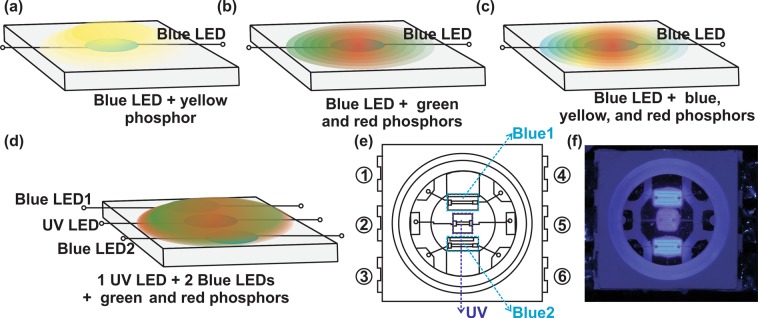
(**a–d**) Schematic showing the structure of the LED PKGs, (**e**) sketch of the LED PKG fabricated using two blue LEDs, and one UV LED, (**f**) digital image of the PKG as shown in (**e**).

Figure 1(a–d) shows the schematic of LEDs fabricated in this study. Figure 1(d,e) show a newly designed PKG structure of width x length x thickness = 5.0 mm × 5.4 mm × 1.2 mm developed to incorporate three LEDs (one UV and two blue LEDs). The mounting area in which the chips were directly mounted was a circular structure with a diameter of 2 mm and a square structure at the center. The main component was an integrated heatsink that directly mounts the chip and absorbs the heat generated by the chip and distributes it to the heat sink.

We fabricated five LED PKGs– (1) blue LED - yellow phosphor (1B-Y), (2) blue LED - green and red phosphors (1B-GR), (3) blue LED - blue, yellow and red phosphors (1B-BYR), (4) and (5) one UV and two blue LEDs - green and red phosphors (PKG-1-1UV2B-GR and PKG-2-1UV2B-GR). Two different PKGs were fabricated using 1UV2B-GR design. In the PKG-1, the wavelength of the blue LED was 452 nm, while in PKG-2, the wavelength of the blue LED was 455 nm. The structure of the final PKG of 1UV2BGR configuration was a 3 in 1- type chip in which a UV chip is placed at the center of the PKG, and two blue chips were arranged on the top and bottom (Fig. 1e). Figure 1f shows the digital image of the LED PKG.

**Figure 2:**
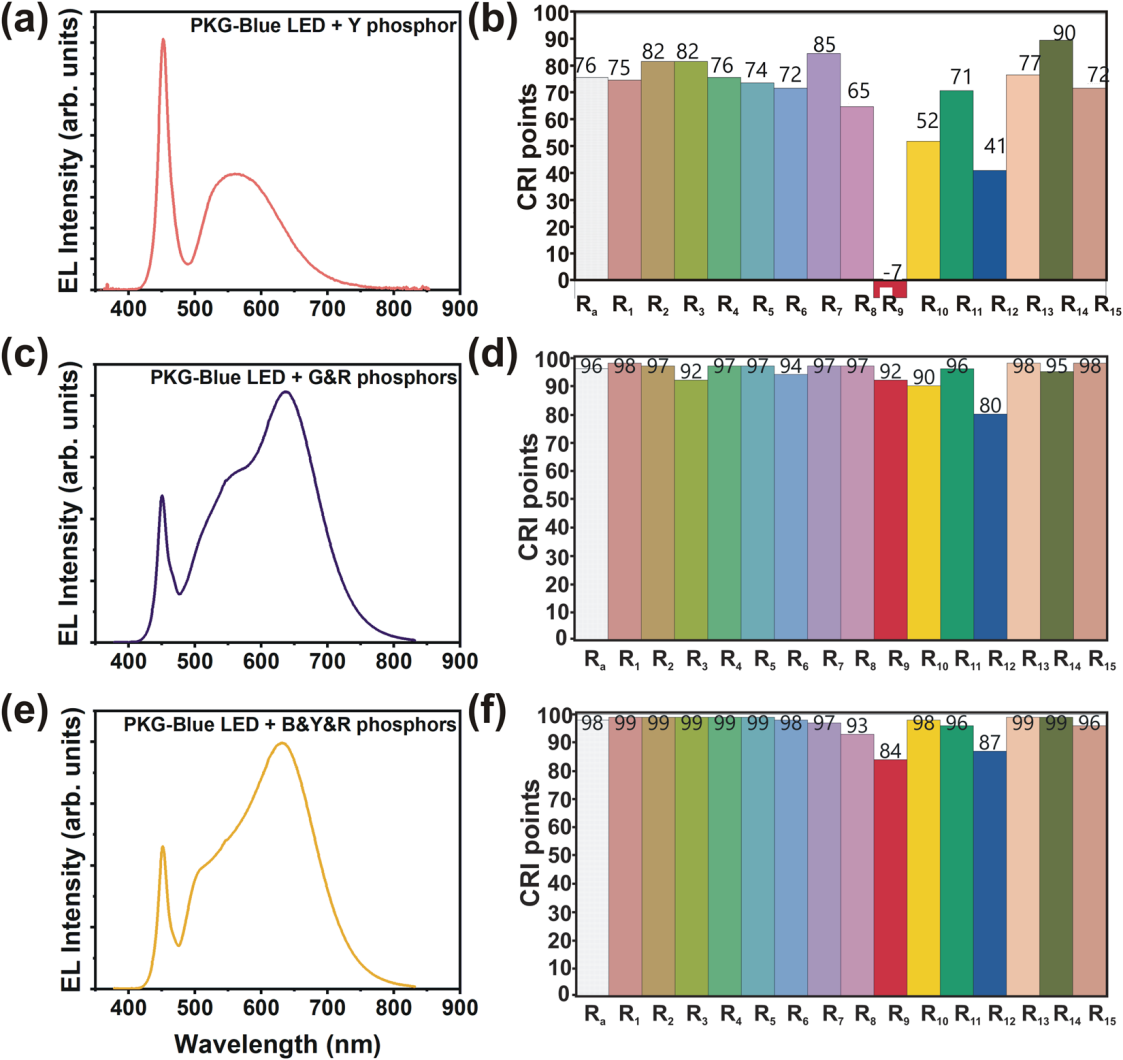
(**a,c,e**) EL emission spectra and (**b,d,f**) the CRI points of the 1B-Y, 1B-GR, and 1B-BYR LEDs.

Figure 2 shows the EL emission spectra and the corresponding CRI points of the conventional Blue LED and phosphor combinations. The 1B-Y PKG has a CRI *R*_a_ of 77 and most of the CRI indices exhibit low values which is unsuitable for athletic stadium lighting and UHDTV production (Fig. 2b). Especially *R*_9_ value is negative (−7), indicating that 1B-Y PKG cannot render the red color accurately because of the lack of red component from the 1B-Y PKG. However, we achieved the highest luminous efficacy of 133 lm/W in this 1B-Y LED PKG because of the trade-off between luminous efficacy and CRI.

For achieving higher CRI, the emission spectrum should be as broad as it can. On the other hand, the broader the emission spectrum, the luminance efficacy of radiation (LER) will be low. The trade-off between CRI and LER is usually represented using the following function that optimizes CRI and LER^25^.

1 $${{\rm{F}}}_{{\rm{\sigma }}}({{\rm{\lambda }}}_{1}\ldots .\,,{{\rm{\lambda }}}_{{\rm{n}}},\,{\Delta {\rm{\lambda }}}_{1}\ldots .\,,{\Delta {\rm{\lambda }}}_{{\rm{n}}},\,{{\rm{I}}}_{1}\ldots \mathrm{..}{{\rm{I}}}_{{\rm{n}}})={\rm{\sigma }}\mathrm{LER}+(1-{\rm{\sigma }}){\rm{CRI}}$$

Where σ corresponds to the weighting factor (0 ≤ σ  ≤ 1), n – number of primary LEDs, λ_1_, λ_2_, Δλ_1_, Δλ_2_, and I_1_, I_2,_ etc. are peak wavelengths, spectral line widths, and the luminous fluxes. Trichromatic white LED lamps are usually used to achieve a balance between CRI and LER. Please note that in Eq. (1) the CRI is CRI R_*a*_. The individual CRI points are not considered in the equation. Furthermore, CRI R_*a*_ can still show very high value even though some of the CRI points are very low. To achieve CRI points above 95 and LER ~ 100 lm/W simultaneously, we fabricated various WLEDs via the combination of the blue LED, UV LED, and phosphors. We investigated how each CRI point changes with the green/red phosphor weight ratio and the current through each LED chip.

In order to enhance the CRI points, firstly, we tried two methods. Since the *R*_9_ value (corresponding to the red component) is negative, we fabricated a PKG with blue LED - green and red phosphors (1B-GR), and the corresponding EL spectrum and CRI points are shown in Fig. 2(c,d). Even though we achieved a high CRI *R*_a_ of 96.2 in this PKG, the *R*_3_, *R*_9_, and *R*_12_ values are still below 95. *R*_12_ represents Munsell code 3PB 3/11, which corresponds to a blue color having an emission peak around 465 nm. To compensate for this, we fabricated a PKG comprising of blue LED - blue, yellow, and red phosphors. Note that the conventional blue phosphor emits light with a peak wavelength of around 490 nm. The EL spectrum and the CRI index points of 1B-BYR LED PKG are shown in Fig. 2(e,f). Again, in this design, we could not achieve all the CRI points above 95. However, it is worth noting that these two LED packages–1B-GR and 1B-BYR, exhibit much better CRI points than 1B-Y PKG. In 1B-GR and 1B-BYR LED PKGs, we tried different combinations of phosphors ratio; however, we could not enhance the CRI points ≥95 along with a luminous efficacy above 100 lm/W. Note that we could achieve CRI points >95; however, the efficacy was lower than 100 lm/W because of the trade-off between CRI points and the efficacy of the PKG. Therefore, in this study, we have designed the PKG by keeping the luminous efficacy around 100 lm/W, and thus, some of the individual CRI points were <95.

To achieve all the CRI indices above 95 and the luminous efficacy >100 lm/W simultaneously, we introduced a UV LED chip along with the two blue LEDs in the PKG. We also fabricated one UV and one blue LED chip configuration. However, because of the low efficiency of the UV LED, the overall luminous efficacy of the PKG was well below 100 lm/W. Therefore, we had to use the one UV and two blue LEDs configuration to enhance efficiency. Note that the integration of three LEDs in a chip results in excessive heat generation. Therefore, we designed the heat sink for efficient dissipation of heat. The strategy to introduce a UV LED resulted in an emission band around 470 nm from the green phosphor, which compensated for low CRI *R*_12_ in the 1B-GR and 1B-BYR LEDs.

To fabricate LED PKG consisting of 1 UV LED, two blue LEDs along with green and red phosphors, we designed two packages.

**Figure 3:**
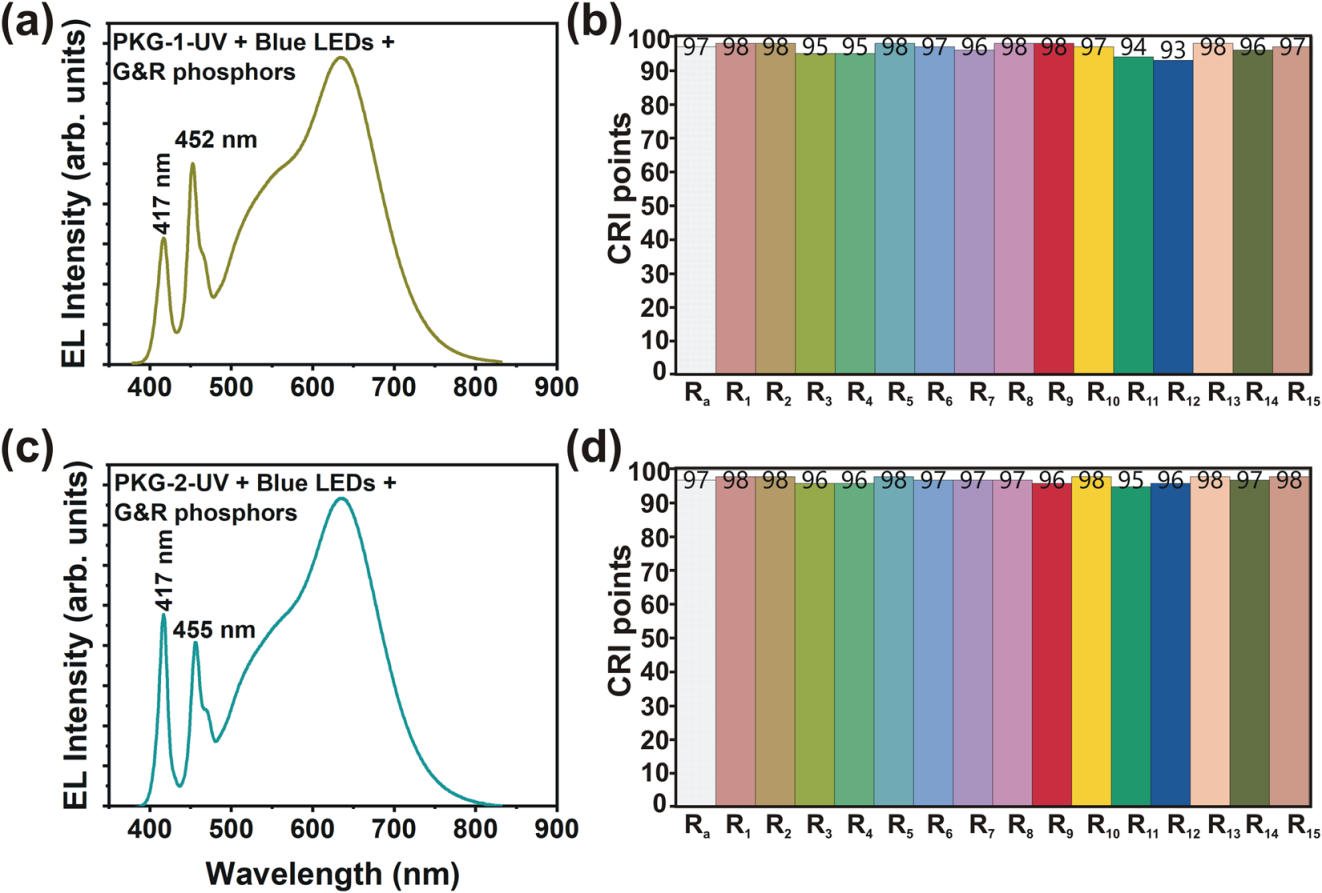
(**a,c**) EL emission spectra and (**b,d**) CRI points of the PKG-1-1UV2B-GR and PKG-2-1UV2B-GR LEDs.

In PKG-1, the wavelength of UV LED was 417 nm, while that of the blue LED was 452 nm. In the PKG-2, we used the same UV LED with a peak emission wavelength of 417 nm. However, we choose the blue LED with a peak emission wavelength of 455 nm. The combination of the 417 nm emission from the UV LED, and 455 nm from the blue LEDs were able to satisfy both the high efficiency of PKG (~ 100 lm/W) and the high color rendering indices (≥95). The EL spectra and the corresponding CRI points are shown in Fig. 3. In PKG-1, the total current was optimized at 60 mA (40 mA through two blue chips and 20 mA through UV chip). In PKG −2, 25 mA (15 mA through two blue chips and 10 mA through UV chip) was set. As seen in Fig. 2(a,c), the emission from UV LED is high in PKG-2. The ratio of the current flowing through UV and blue LEDs was optimized to achieve a luminous efficacy of ≥100 lm/W.

Note that the *R*_11_ and *R*_12_ values were below 95 for the PKG-1-1UV2B-GR LED PKG because the emission wavelength of the blue-chip was around 452 nm. The blue-chip used in the PKG-2-1UV2B-GR LED PKG emits light with a peak wavelength of 455 nm. It is because of the peak emission of 455 nm from this blue-chip; we could achieve all the CRI points >95.

**Figure 4:**
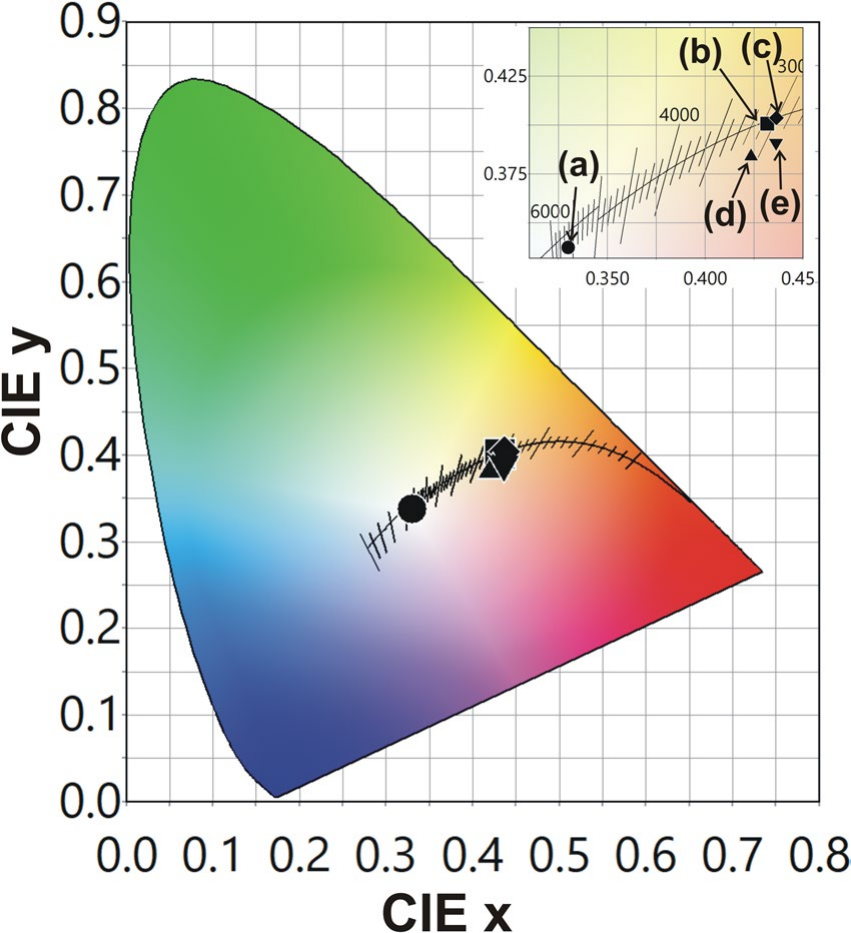
CIE 1931 color coordinates and the corresponding CCT values of (**a**) 1B-Y, (**b**) 1B-GR, (**c**) 1B_BYR, (**d**) PKG-1-1UV2B-GR and (**e**) PKG-2-1UV2B-GR

Figure 4 shows the CIE (x,y) coordinates and correlated color temperatures (CCT) of the WLEDs fabricated in this study.

Each coordinate in the CIE 1931 chromaticity diagram represents the CCT value and the coordinates of CIE (x, y), respectively. The CCT and CIE (x, y) coordinates of each PKG are summarized in Table 1. In the inset of Fig. 4, an enlarged view is shown. (a), (b) and (c) are the color coordinates and CCT of 1B-Y, 1B-GR, and 1B-BYR LED PKGs and, (d), (e) represents the corresponding coordinates of PKG-1-1UV2B-GR and PKG-2-1UV2B-GR LEDs respectively. When compared to 1B-Y LED PKG, all other LEDs exhibit much warmer emission because of the contribution of the red component in the 1B-GR, 1B-BYR, PKG-1-1UV2B-GR, and PKG-2-1UV2B-GR LEDs. Moreover, the CIE coordinates of PKG-1-1UV2B-GR and PKG-2-1UV2B-GR LEDs lie below the black body locus. Studies suggest that the many observers prefer sources with the CIE coordinates below the blackbody locus^26–28^. The preference to the sources with the CIE coordinates below the blackbody locus is because of the combined effects of chromaticity and color rendering effects. Studies based on simulations reported that the light sources with chromaticity below the blackbody locus are more likely to have higher scores for relative gamut while maintaining high scores for fidelity.

**Table 1:** The CRI *R*_a_, *R*_9_, CIE(x,y) coordinates, CCT values and luminous efficacy of the fabricated LEDs.

	CRI (Ra)	CRI (R9)	CIEx	CIEy	CCT (K)	lm/W
1B-Y	76	−7	0.3300	0.3375	5608	133.00
1B-GR	96	92	0.4318	0.4007	3060	106.20
1B-BYR	98	84	0.4365	0.4038	3006	105.71
PKG-1 1UV2B-GR	97	98	0.4234	0.3844	3075	107.31
PKG-2 1UV2B-GR	97	96	0.4363	0.3916	2906	100.12

As discussed before, the CRI *R*_9_ of 1B-Y LED PKG was −7, indicating that the 1B-Y LED PKG has poor or no red color rendering ability. As the CRI points are scaled between 0 and 100, a negative CRI point does not provide any information. If two WLEDs have different negative CRI points, then it is impossible to compare the particular color rendering ability. CRI R_*i*_ is calculated using the following equation^17,25^

2 $${R}_{i}=100-4.6\,\Delta {E}_{i}$$

where ΔE is the shift in the color/color differences. If ΔE ≥ 22, then *R*_i_ will show negative values.

**Figure 5:**
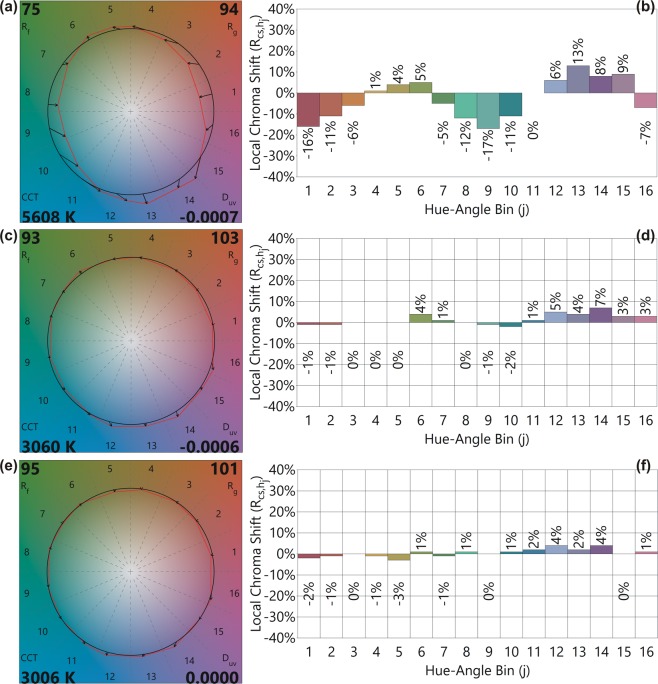
(**a,c,e**) TM-30-18 color vector graphics (CVGs) and (**b,d,f**) local chroma shifts of the 1B-Y, 1B-GR, and 1B-BYR LEDs. A black circle shows the reference illuminant, and the red circles represent the CVGs of the fabricated LEDs. The arrow indicates the corresponding color change induced by 16 hue bins compared to the reference illuminant.

Therefore, only CRI R_*i*_ cannot be used to evaluate the fabricated LEDs, and the LEDs were further assessed using the TM-30 standard method. In the TM-30 standard, 99 color samples are used to evaluate the light source’s performance. Except for the 1B-Y LED, all other LED PKGs exhibit high color fidelity in TM-30 standard. Figure 5(a,c,e) show color vector graphics (CVGs) and Fig. 5(b,d,f) show the local chroma shifts of the light emitted from the 1B-Y, 1B-GR, and 1B-BYR LEDs (red circles) compared to the reference illuminant (black circles). The arrows in Fig. 5(a,c,e) indicate the shift of the 16 hue bins compared to those of the reference illuminant.

As evident from the CVGs, the 1B-Y LED PKG exhibit a decrease in all the 16 hue bins, whereas the 1B-GR and 1B-BYR LED PKGs to exhibit much lesser shifts from those of the reference illuminant. The Chroma shifts indicate the relative percentage (positive/negative) shifts of the 16 hue-angle bins. The CVG of 1B-Y LED show that the light source fabricated using 1B-Y LED will result in less saturated images. As expected, the 1B-Y LED PKG exhibit a significant shift in the local Chroma values. For 1B-Y LED PKG, most of the Chroma values show a negative shift, and the change up to −17% is observed.

On the other hand, the CVGs of 1B-GR and 1B-BYR exhibit a lesser shift of the 16 hue bins. The red circles (our LED) almost overlap with the black one (reference illuminant). The local Chroma shifts for the 1B-GR and 1B-BYR were under 7% and 5%, respectively. The D_uv_ – a measure of the CCT value shift from the black body locus is −0.0007 for 1B-Y LED PKG. The D_uv_ of the 1B-GR is −0.0006 and 0 for 1B-BYR. Note that the CCT value of the 1B-BYR LED PKG is 3006, which lies almost on the black body locus, and therefore there is no shift from the black body locus.

**Figure 6:**
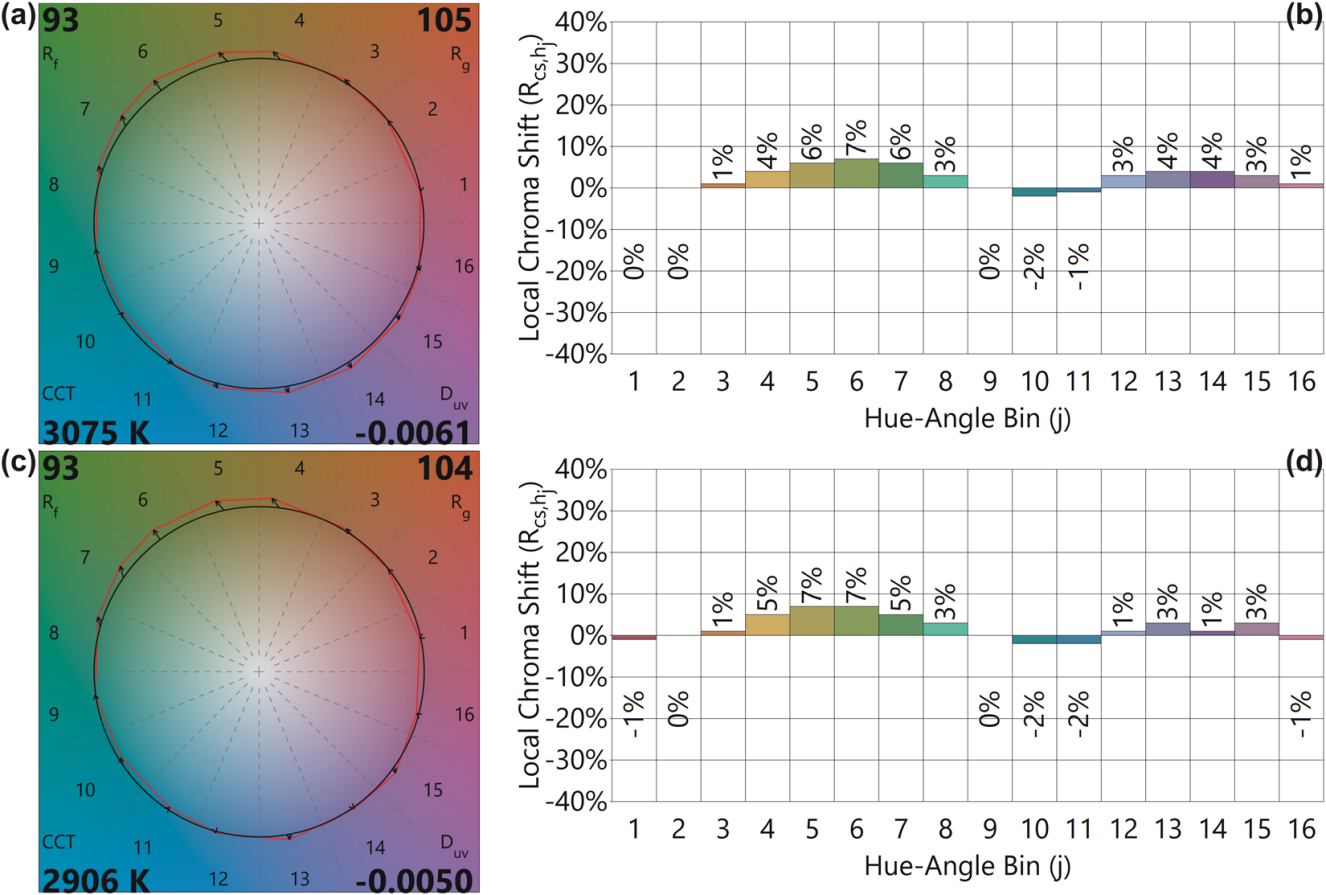
(**a,c**) TM-30-18 color vector graphics (CVGs) and (**b,d**) local chroma shifts of PKG-1-1UV2B-GR and PKG-2-1UV2B-GR LEDs. A black circle shows the reference illuminant, and the red circles represent the CVGs of the fabricated LEDs. The arrow indicates the corresponding color change induced by 16 hue bins compared to the reference illuminant.

The CVGs and the local Chroma shift of the PKG-1-1UV2B-GR and PKG-2-1UV2B-GR LEDs are shown in Fig. 6. The local Chroma shifts for these LEDs are under 7%, mostly positive shifts. The D_uv_ values are −0.0061 and −0.0050 for PKG-1-1UV2B-GR and PKG-2-1UV2B-GR LEDs, respectively indicating that these light sources will be highly preferred.

**Figure 7:**
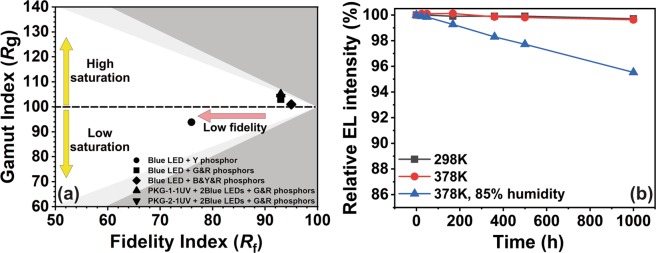
(**a**) IES TM-30-18 Fidelity index (*R*_f_) and gamut index (*R*_g_) of the fabricated LEDs. Light grey region – Approximate limits for sources on the Planckian locus, Dark grey area – Approximate limits for practical light sources. (**b**) The thermal stability of the PKG-2-1UV2B-GR LED under stress conditions–298 K, 378 K, and (378 K, 85% humidity).

Figure 7a shows the *R*_g_ vs. *R*_f_ plot of the fabricated LEDs. The ideal value of the (*R*_g_, *R*_f_) is (100, 100). The deviation of the values indicates whether the light source produces under/over-saturated images or low fidelity images. The 1B-Y LED PKG exhibits low R_g_ and R_f_ values indicating the LED produces low fidelity and low-saturated images. All the other LED PKGs exhibit reasonable (R_g_, R_f_) values. The optimum value was achieved for the 1B-BYR LED PKG, which has (R_g_, R_f_) values of (95, 101) and produces images with high fidelity and nearly perfect saturation. Note that 1B-BYR LED PKG did not have all the CRI points above 95. The PKG-1-1UV2B-GR and PKG-2-1UV2B-GR LEDs can be used to produce images with high fidelity and high saturation because of its high (R_g_, R_f_) values.

It is worth noting that the *R*_f_ value is lower than the CRI (*Ra*) value in all the LED PKGs because the TM-30 standard uses 99 color samples, whereas CIE *R*_a_ uses only 8 sample colors. Therefore, R_f_ can be lower than *R*_*a*_. Finally, the long term stability and reliability of the newly designed PKG-2-1UV2B-GR LED was tested under various test environments. Figure 7b shows the reliability data. We tested the thermal stability/ reliability of the LEDs by keeping it ON at 298 K, 378 K, and 378 K, 85% humidity for 1000 h. The emission intensity was 99.7% of the initial value even after 1000 h of operation. Besides, we tested the sample under 378 K, and a current of 180 mA was applied to measure the change of the luminous flux up to 1000 h, and the luminous emission intensity of 99.6% of the initial emission intensity was maintained even after 1000 h of continuous operation. Finally, the PKG-2-1UV2B-GR LED was kept in a chamber having a temperature of 378 K and a humidity of 85%, and the durability under stress conditions was confirmed. The PKG-2-1UV2B-GR LED exhibited a high luminous intensity of 95.5% of the initial emission intensity, suggesting that the fabricated LEDs are highly reliable and durable even under stress conditions.

Finally, we evaluated the power consumption of our newly designed configuration. It is worth pointing that when compared to the conventional 1B-Y LEDs, our 1UV2B-GR LEDs consume less power, even our 1UV2B-GR LEDs consist of one UV and two blue LEDs. The power consumed by our 1UV2B-GR LEDs is 69 mW, which is slightly lower than the power consumed by conventional 1B-Y LEDs (71.3 mW). The power consumption was calculated when both the LED PKGs were operated under a forward current of 25 mA. The newly designed LED PKGs are having a high efficacy of 100 lm/W, high CRI points above 95, and high durability will find applications in artificial stadium lighting, UHDTV production, and applications where highly efficient and high color rendering WLEDs are required.

## Conclusion

To utilize the WLEDs for the application that requires sunlight-like quality, LED PKG with CRI (*R*_1_-*R*_15_) points exceeding 95 were fabricated using a re-designed LED PKG consisting of one UV LED, two blue LEDs and green & red phosphors. The fabricated LED PKGs not only exhibit CRI *R*_1_-*R*_15_ points above 95 but also have a luminous efficacy above 100 lm/W. We achieved high efficiency and CRI points above 95 through adjusting the peak emission wavelength of UV and blue LEDs, current ratio through the LEDs, and the green/red phosphor mixing ratios. An optimized CRI Ra of 97.3 with an efficiency of 100.12 lm/W and CCT of 2906 K was achieved from the PKG-2-1UV2B-GR LED. The fabricated LEDs were evaluated using TM-30-18 color vector graphics, local Chroma shifts, and fidelity-gamut plot. The newly designed LED PKGs exhibit high fidelity and high saturation values. The LEDs also exhibit high stability and reliability, exhibiting a stable operation up to 1000 h under 378 K, 85% humidity test conditions. Our study on the re-designed LED PKG consisting of one UV LED, two blue LEDs - green and red phosphors paves the way to fabricate and use WLEDs in stadium lighting and UHDTV production where high CRI points, high efficiency, and low power consumption are required.

## Experiment

### LED PKG design

In this study, eight middle size chips of each LED chip manufacturer were selected and compared with each other. The test LEDs were fabricated in this study using various LEDs developed by multiple companies. Considering the luminous efficacy and CRI points, we have used 2140 LED chips in this study. In order to realize high CRI and improve the light efficiency of the PKG, six power terminals (two for each LED) were used to control the currents of the bonded chips individually. Also, the PKG includes an integral heatsink for absorbing heat generated from the chip and dispersing the heat into a heat radiator and a terminal for applying an external power source. The PKG designed in this study is characterized by the structure of a single encapsulation for phosphor that can bond blue and UV chips simultaneously and phosphors that can absorb the excitation light of two wavelengths (UV and blue).

### Selection for silicone encapsulant

In the LED PKG, the role of an encapsulant is to protect the chip and the bonding wire inside the PKG from the physical impact or external environment. Additionally, it serves as a medium to emit light generated from the UV/blue-chip as well as the phosphor. Silicone encapsulant for protecting chips and wire has a double reflector structure for stable filling, and a high heat resistant resin was used for achieving high reliability. In this newly designed PKG structure, we made a silicone resin by mixing pure silicone and phenyl-based silicone, and the content and mixing ratio of green and red phosphors were optimized to produce PKG having all the CRI points equal to or above 95. The essential characteristics that the encapsulant used for the LED PKG should have are physically resistant to the high heat generated at the UV/blue LED junction, and the interfacial adhesion must be ensured to protect LED from the external high temperature and high humidity environment. To ensure the reliability of the PKG and to maximize the efficiency of light extraction, phenyl-based silicone was used as an encapsulant. The encapsulants used for fabricating LEDs and their properties are summarized in Table 2.

**Table 2:** The physical properties of silicone encapsulants analyzed in the present study.

Silicone encapsulant	OE6370M	OE-6631	OE-7651N
Refractive Index	1.41	1.54	1.55
Mixing ratio (A: B)	1:1	1:2	1:4
Cure condition	150 °C/4 hr	150 °C/1 hr	150 °C/2 hr
Viscosity (Part A) (cps)	5,848	5,500	3,000
Viscosity (Part B) cps	2,000	13,500	3,300
Viscosity (mixed) (cps)	3,308	7,150	3,300
Hardness shore	A71	A60 ~ A69	D51
Elongation @ RT%	80	57	120
Modulus @−40 °C (MPa)	3	1,264	1,290
Modulus @25 °C (MPa)	2.3	7.8	18.0
Modulus @125 °C (Mpa)	3.1	0.1	0.8
Tensile strength @ RT (Mpa)	9.2	2.4	4.8
Permeability_O_2_ (cm^3^/m^2^/24 h/atm)	20,000	880	420
Permeability_H_2_O (g/m^2^/24 h)	100	22	10
Transmittance % @450 nm	100	100	95
Shelf life at 25 C (Days)	360	360	360

Because of less oxygen and water permeability of OE-7651N encapsulant, we have used OE-7651N for fabricating LEDs.

### Materials

The yellow phosphor used in this study is commercial YAG:Ce^3+^ phosphor. The green and red phosphors are GNYAG3757 and R6634 developed by Intematix. In the 1B-GR LED package, the optimized ratio of green and red phosphors was 14% and 2.7%. In the 1B-BYR LED package, the ratio of blue, yellow, and red phosphors were 4%, 6%, and 2.3%, respectively. Two blue LED chips were used in the study, with the peak emission wavelengths around 452 and 455 nm. Table 3 shows the various chips analyzed for the present study. PKGs were fabricated using the chips to achieve the required CRI. After careful evaluation, Corp.C’s 2140 chip was used to make 1UV2B-GR LEDs.

**Table 3:** Power output and emission bands of the blue chips manufactured by various companies.

Maker	Size*	P_0_ [mW]	Width [nm]
Corp. A	4222	150.0–155.8	455.1–456.4
Corp. A	2432	120–121.5	455.0–457.5
Corp. B	4422	145.8–149.9	452.6–454.9
Corp. C	2646	230–260	450–452.5
Corp. C	2140	170–180	455–457.5
Corp. D	4022	225.3–229.9	455–457.5
Corp. E	2235	170–180	455–457.5
Corp. E	2445	180–190	455–457.5

^*^4222 corresponds to 4.2 × 2.2 mm^2^.

### Characterization of the fabricated LEDs

We evaluated the efficiency of the chip by recording the EL spectra using CAS 140CT (Instrument Systems, Germany) and 500 mm integrating sphere. The forward current to the LEDs was applied using the constant current source K2425 (Keithley). The output characteristics of the fabricated LEDs were evaluated using the CIE and TM-30-18 standards.

## Data Availability

The datasets generated during and analyzed during the current study are available from the corresponding author on reasonable request.
